# Replace or Regenerate? Diverse Approaches to Biomaterials for Treating Corneal Lesions

**DOI:** 10.3390/biomimetics9040202

**Published:** 2024-03-28

**Authors:** Pietro Bonato, Andrea Bagno

**Affiliations:** Department of Industrial Engineering, University of Padua, 35131 Padua, Italy

**Keywords:** cornea, keratoplasty, keratoprosthesis, cornea regeneration, cornea transplantation, bioprinting, xenotransplantation, 3D bioprinting

## Abstract

The inner structures of the eye are protected by the cornea, which is a transparent membrane exposed to the external environment and subjected to the risk of lesions and diseases, sometimes resulting in impaired vision and blindness. Several eye pathologies can be treated with a keratoplasty, a surgical procedure aimed at replacing the cornea with tissues from human donors. Even though the success rate is high (up to 90% for the first graft in low-risk patients at 5-year follow-up), this approach is limited by the insufficient number of donors and several clinically relevant drawbacks. Alternatively, keratoprosthesis can be applied in an attempt to restore minimal functions of the cornea: For this reason, it is used only for high-risk patients. Recently, many biomaterials of both natural and synthetic origin have been developed as corneal substitutes to restore and replace diseased or injured corneas in low-risk patients. After illustrating the traditional clinical approaches, the present paper aims to review the most innovative solutions that have been recently proposed to regenerate the cornea, avoiding the use of donor tissues. Finally, innovative approaches to biological tissue 3D printing and xenotransplantation will be mentioned.

## 1. Introduction

**Figure 1:**
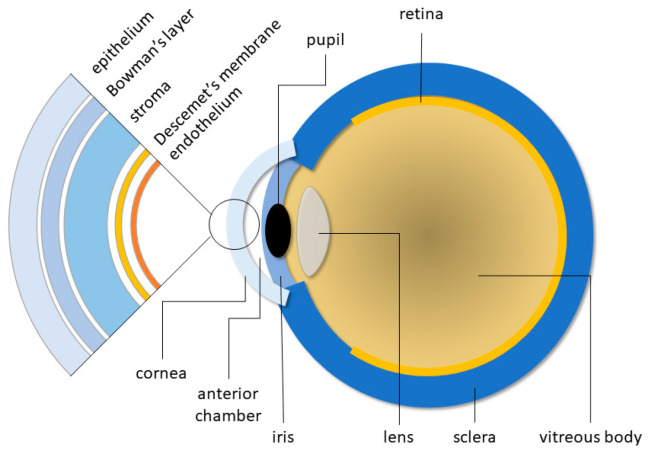
Schematic diagram of the human eye, highlighting the cornea structure.

The cornea is a convex, transparent, non-vascularized tissue that covers and protects the pupil, iris, and the anterior chamber of the eye (Figure 1); it allows for generating vision by transmitting the light to the back of the eye. It is about 12 mm in diameter, 0.54 mm thick in the central part, and 0.7 mm at the edges; the difference in thickness is due to the more abundant presence of collagen in the stroma. The inner curvature radius is about 8 mm.

Besides its protective role, the cornea allows light to reach the retina, providing approximately 40–44% of the refractive power. A high refractive index and a symmetrical curvature are both optimal for maximum refractive power and minimum astigmatism [1]. The innervation of the cornea is 300–600 folds of the skin and 20–40 folds of the gum, making it one of the most innervated tissues of the human body [2]. Many are the sensory nerves originating from the ophthalmic branch of the trigeminal nerve: They play an important role in sensory functions, in the maintenance of homeostasis of the corneal epithelium, in the production of tears, allowing movements such as blinking the eyelashes [3].

The human cornea is composed of three cellular layers: the outer corneal epithelium, the intermediate stroma, and the inner endothelium. These layers are separated by the Bowman’s lamina (between epithelium and stroma) and the Descemet’s membrane (between stroma and endothelium) [4].

The epithelium is made of squamous non-keratinized cells. In the center of the cornea, there are 5–7 cellular layers, while over 10 layers are located in the peripheral areas [2,5]. The thickness is approximately 40 µm in the center and increases at the edges (50 µm). It is the outermost layer of the cornea, being continuously exposed to the external environment. It can be divided into three sublayers: superficial, intermediate, and basal. A total of 2–3 layers of cells are located on the surface: They prevent toxins, microorganisms, and tears from entering the eye. Once a week, the epithelium is totally renewed by means of the centripetal migration of the stem cells present in the corneal limbus [2], which is a ring of tissue in the peripheral area of the cornea in direct contact with the sclera [5]. Cell differentiation occurs simultaneously with their migration, passing from basal cells to intermediate cells and finally to superficial cells [2]. The epithelium is in a symbiotic relationship with the overlying tear film. The mucous layer of the tear film in direct contact with the corneal epithelium is produced by goblet cells: Their close interaction allows for diffusion of the tear film at every blink [1].

The Bowman’s layer is a condensed acellular layer in the apical region of the stroma: It mainly contains collagen types I, III, V, VI, and XII, but also collagen types IV and VII, coming from the lower membrane. Collagen is organized into interconnected fibrils: It accounts for a thickness of 8–12 µm that decreases with aging and is unable to regenerate after injury or surgical removal [2]. Its elasticity helps the cornea maintain its typical shape [1]. Being resistant to trauma and infection, it acts as an additional barrier for the inferior layers, also regulating the transfer of molecules [2].

The stroma represents a major part of the cornea: With a thickness of 500 µm, it covers 80-85% of the cornea [2]. It is composed of well-aligned collagen fibers, proteoglycans, and stroma cells, i.e., keratocytes [6]. It mainly contains collagen types III, VI, XII, and XIV, surrounded by proteoglycans associated with glycosaminoglycans. The precise three-dimensional arrangement of fibers and cells, with the presence of chondroitin sulfate and keratan sulfate, is responsible for the transparency of the cornea and its hydration [2]. The well-defined and parallel collagen bundles are called lamellae. The human eye contains 200–250 lamellae approximately [1]. Unlike corneal epithelial cells, stroma cells cannot self-regenerate: They remain in a quiescent state. Due to diseases or injuries, keratocytes will transform into fibroblasts able to secrete new extracellular matrix for stroma regeneration [5]. If the lesion is too large to spontaneously heal, or some cells are damaged, scars can be formed, interfering with the normal hydration of the cornea and causing it to lose its typical transparency [6].

The Descemet’s lamina is a basal layer constantly secreted by the corneal endothelium: It maintains the structure of the endothelium itself. Its thickness is of about 3 µm at birth, but, unlike the Bowman’s lamina, it increases over time up to 10 µm. The anterior part of the lamina, produced before birth, is fibrous; the posterior part, produced after birth, is more homogeneous. The lamina is essentially composed of collagen types III, V, and VII, but traces of fibronectin and laminin can also be found. An important function is its co-participation in the hydration of the cornea together with the endothelium [2].

The corneal endothelium is organized into a single layer of hexagonal cells covering the back of the cornea. The endothelial cells, linked to each other via tight junctions, form a 5 µm thick layer in direct contact with the underlying aqueous humor via hemidesmosomes. Unlike corneal epithelial cells, endothelial cells are not capable of mitosis and proliferation. Damaged cells are replaced thanks to the centripetal migration of peripheral endothelial cells [2]. Cell density is one of the most important factors for endothelial function [6]: Cell density decreases with age and, since the endothelial cells cannot replicate, the surface area covered by each cell has to enlarge [2]. The cornea lacks blood vessels: Thus, the endothelial cells are responsible for maintaining the deturgescence of the cornea by means of sodium–potassium pumps located in the lower portion of the membrane. These pumps passively transfer ions and water from the hypotonic stroma to the hypertonic aqueous humor. This mechanism is crucial to maintain the transparency of the cornea, while ions and water exchanges allow for the passage of nutrients [2].

Being located in the outer part of the eye, the cornea is permanently exposed to a number of stimuli (i.e., abrasion, mechanical and chemical forces, temperature variations), which can be the reasons for corneal lesions [2]. Corneal diseases represent the fifth cause of blindness in the world; they can be due to infections, autoimmune reactions, dysregulation of the tear film, and corneal decompensation. Infectious diseases of the cornea mainly derive from bacteria, fungi, and viruses. According to the World Health Organization (WHO), 285 million people worldwide suffer from impaired vision, and among these, 39 million are blind [5].

## 2. Lesions and Diseases Affecting the Cornea

**Figure 2:**
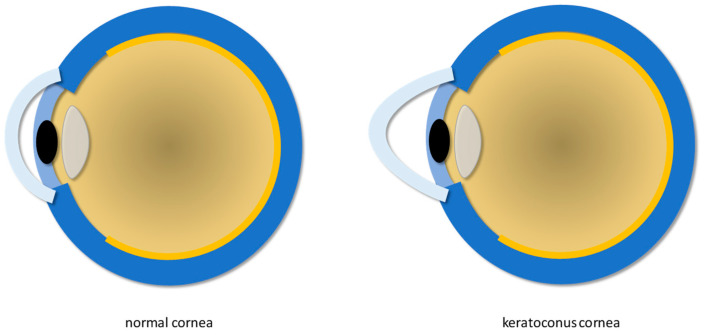
Normal cornea (**left**) and keratoconus cornea (**right**).

Infection due to Herpes Simplex Virus (HSV), keratoconus (Figure 2), and endothelial dystrophies are some of the most common corneal diseases.

HSV is a neurotropic virus that can infect the skin, mouth, mucous membranes, genitals, and eyes [7,8]. It is considered one of the primary causes of corneal diseases and vision loss. In particular, HSV serotype 1 is commonly associated with corneal infection [5]. When the reactivation of latent HSV-1 involves the stroma and endothelium more significantly, it can result in Herpes Simplex Keratitis (HSK) [5,7]. Clinical signs of HSK include stromal opacity, edema, neovascularization, and linear corneal dendritic lesions with branching of terminal bulbs [9,10,11]. Cases involving the stroma account for approximately 20–48% of recurrent HSV infections, leading to long-term vision loss due to corneal scarring. Clinical manifestations include stromal infiltrations, which can lead to stromal edema, corneal thinning, and widespread stromal neovascularization [12].

Keratoconus is a bilateral non-inflammatory ectasia with an incidence of approximately 1 case out of 2000 individuals worldwide, with even higher estimations in some geographic areas due to specific genetic characteristics and possible favorable environmental conditions [3]. The diagnosis of keratoconus includes the following: keratoglobus (a rare non-inflammatory disorder characterized by generalized thinning and globular protrusion of the cornea [13]); pellucid marginal degeneration (a non-inflammatory ectatic corneal disease characterized by a narrow band of thinned cornea separated from the corneal limbus by a relatively uninvolved area of 1–2 mm in width [14]); Terrien’s marginal degeneration [15] (a rare disease manifesting with decreased visual acuity due to increased corneal astigmatism [16]). Thinning of the corneal epithelium around the apex of the cone and the conical shape of the cornea are considered the most common histopathological changes associated with keratoconus [15,17]. It has been reported that in keratoconus, the apical region of the cornea shows a reduction in the number of lamellae, which is correlated with the severity of the disease [18,19]. It is worth underlining that keratoconus, pellucid marginal degeneration, and Terrien’s marginal degeneration can be all classified as corneal disorders that involve thinning and distortion of the cornea; indeed, they share the same similar symptoms, but they possess distinct characteristics. For instance, keratoconus mainly affects the central (paracentral) part of the cornea; pellucid marginal degeneration mostly occurs in the lower part of the cornea with the typical “butterfly wing” shape; Terrien’s marginal degeneration starts from the marginal part of the cornea and progresses to the central part. Moreover, the cone-shaped cornea caused by keratoconus results in an irregular astigmatism; pellucid marginal degeneration causes a steepening effect in the lower half of the cornea; Terrien’s marginal degeneration is characterized by the development of a band-like thinning. An accurate diagnosis is crucial for appropriate management and treatment.

Finally, corneal dystrophies, usually bilateral and symmetrical, are among the most common dysfunctions affecting endothelial cells. They cause morphological damage to endothelial cells, disrupting the activity of sodium–potassium pumps, causing corneal edema, bubbles, and subepithelial fibrosis [20]. The most common dystrophy is Fuch’s dystrophy, accounting for approximately 39% of corneal transplants [21]. Although Fuch’s dystrophy primarily affects the corneal endothelium, the loss of endothelial cells can lead to changes in the Descemet’s membrane, accumulation of extracellular matrix, and formation of posterior focal outgrowths called guttae [22].

## 3. Current Therapeutic Approaches

### 3.1. Keratoplasty

Over the years, keratoplasty has proven to be the most successful transplant procedure for the treatment of several eye diseases [7,23]: It still remains the primary approach for visual rehabilitation once diseases have damaged the cornea [24]. It achieves approximately a 90% success rate for the first graft in low-risk patients at 5-year follow-up, while patients for whom the first graft was unsuccessful have a 50% success rate for a subsequent re-graft [25].

The only worldwide available data on keratoplasty come from the annual statistical reports of the Eye Bank Association of America, the European Eye Bank Association, and the Association of Eye Banks of Australia and New Zealand, representing less than 15% of the world’s population [23]. 

**Figure 3:**
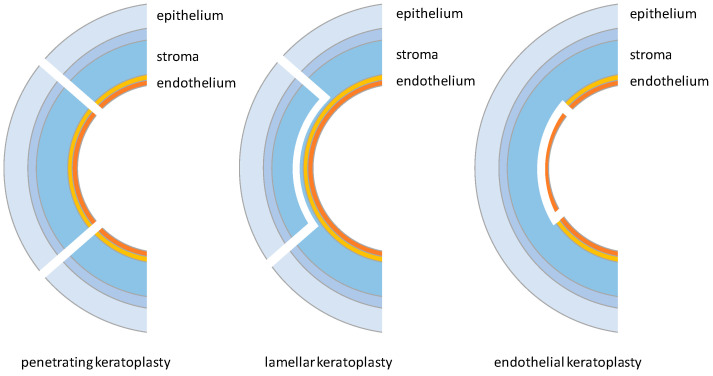
Different types of keratoplasty: penetrating, lamellar, and endothelial.

There are different types of keratoplasty, grouped into three main categories depending on the tissue removed: penetrating keratoplasty, lamellar keratoplasty, and endothelial keratoplasty (Figure 3) [2].

Penetrating Keratoplasty (PK), currently representing 34% of corneal transplants, has become one of the most performed corneal transplant operations, capable of completely replacing the diseased cornea with a healthy one from a donor [2,23,26]. The procedure requires the removal of the cornea through a partial perforation with a drill, followed by the completion of the excision along the cuts made by the drill. In the second part of the procedure, the healthy cornea is placed and fixed to the receiving bed by applying approximately 16 sutures around the cornea with a 10-0 nylon thread [26].

A second technique is gaining growing popularity due to its benefits: Anterior Lamellar Keratoplasty (ALK). An ALK selectively removes the corneal stroma without harming other tissues. Compared to a PK, it offers several additional advantages, including a lower risk of rejection, lower loss of endothelial cells, longer-lasting grafts, and fewer intraoperative and postoperative complications [27,28]. Among current ALK approaches, the Deep Anterior Lamellar Keratoplasty (DALK) has been proposed as an excellent alternative to PKs: Indeed, it is the most widely practiced ALK. Recent DALK surgical techniques involve manual dissection close to the Descemet’s membrane or injection of air, fluids, or viscoelastic materials into the stroma to create a plane that separates the stroma from the Descemet’s membrane [28,29]. In this case, once the tissue is removed, suturing is carried out as in the normal PK. Using this technique, the success rate exceeds 95% with minimal complications [29].

Finally, endothelial keratoplasties (EKs) account for approximately 54% of the procedures performed annually [2], exploited by surgeons worldwide as an alternative to PKs for the treatment of endothelial corneal disorders [30]. An EK involves the selective removal and replacement of the corneal endothelium [31] when only the endothelium is damaged. Compared to a PK, the major benefit is the possibility of maintaining the structural integrity of the eye, thereby reducing the risk of rejection. Other advantages include a more effective and faster visual rehabilitation, minimization of induced astigmatism, and the elimination of some complications due to the final sutures [30,32,33]. This is evidenced by the decreasing annual number of PK procedures over the years in the United States, from 42,063 in 2005 to 19,294 in 2014. Meanwhile, the annual number of EK procedures is continuously increasing, with a total of 25,965 procedures performed in 2014 [34]. Currently, among available endothelial keratoplasties, the Descemet’s Membrane Endothelial Keratoplasty (DMEK) is the most physiological and widely used method to remove endothelial diseases [33]. This approach involves the detachment of the Descemet’s membrane from the posterior stroma using a method similar to that for the ALK. However, its use is limited due to donor preparation problems, such as Descemet’s membrane rupture and the difficulty of deploying healthy tissue into the anterior chamber [35].

### 3.2. Keratoprosthesis

**Figure 4:**
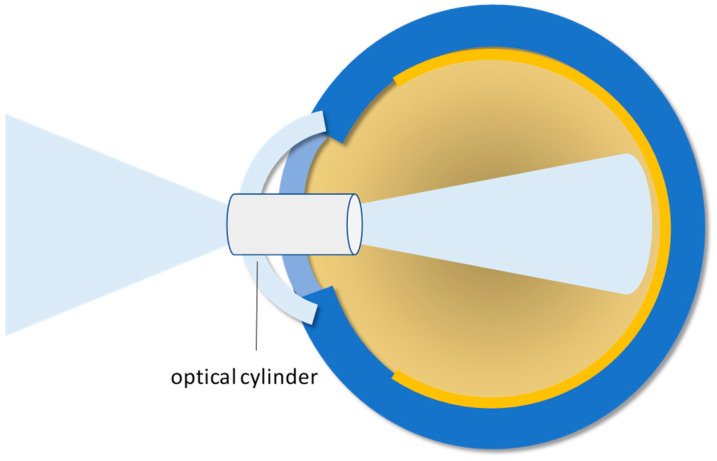
Schematic illustration of the keratoprosthesis: The optical cylinder is the key component allowing the replacement of the minimal functions of the cornea.

When a diseased/injured cornea needs to be replaced, the native tissue is removed and a healthy cornea from a human donor is surgically applied. Although the overall success rate of this approach is up to 85–90%, it inevitably decreases in high-risk cases. There are many complications deriving from keratoplasties: damages to sensory innervation, differences in size between donor and recipient, astigmatism, reduced quality of vision, ineffective healing of surgical wounds, inflammation, infection, neovascularization, and eventually graft rejection. Selective removal of individual corneal layers cannot be performed when the full thickness of the cornea is damaged. An alternative solution for corneal lesions is represented by the keratoprosthesis (KPro), which replaces the minimal functions of the cornea, i.e., the central vision, and protects the internal elements of the eye (Figure 4). Many of these prostheses are available clinically, but their use is not widespread. Since they are proposed as the only solution for high-risk patients, the biointegration of these devices is not effective, thus obscuring the success rates. A different and simpler approach is therefore needed, which can be provided by regenerative medicine (see Section 4).

At present, two models of Boston keratoprosthesis are clinically available: Basically, they differ in the anterior extension of the implant into the eye when the eyelids close. Since 1992, when the Boston-KPro type I was FDA-approved, it has become the most used KPro with more than 15,000 devices implanted worldwide. On the other hand, the Boston KPro type II is less popular: It is indicated for patients with serious ocular diseases, representing the last option to save some vision functions [36,37,38].

**Figure 5:**
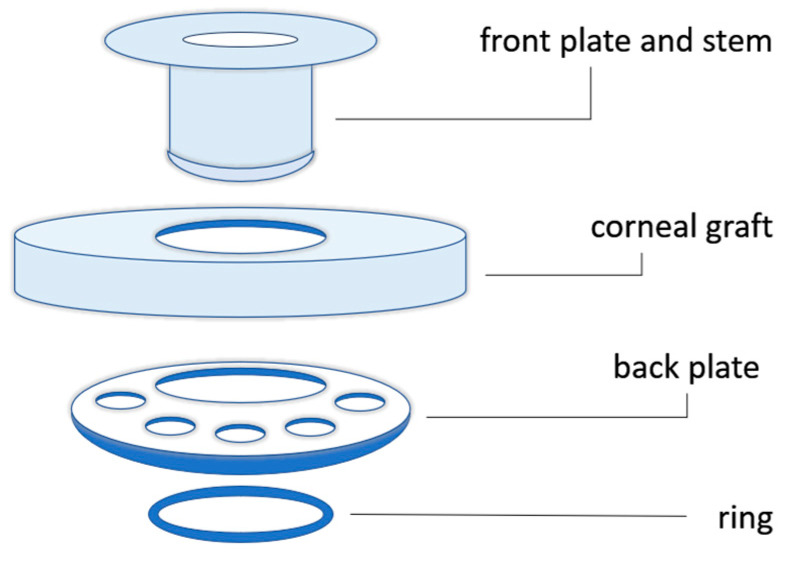
Schematic illustration of the components of the Boston keratoprosthesis type I.

**Figure 6:**
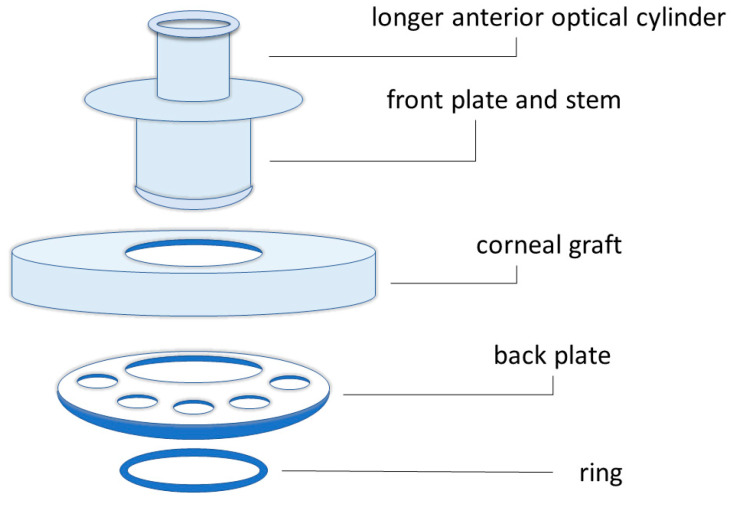
Schematic illustration of the components of the Boston keratoprosthesis type II.

The Boston KPro type I was first introduced by Dr. Claes H. Dohlman in 1970 (Figure 5) [39]. The device consists of a cornea combined with an optical stem and a posterior fixation plate [40]. The type II Boston keratoprosthesis is similar [37], but it has an anterior protuberance that can cross the eyelid or the buccal mucosa (Figure 6) [40].

It is important to point out that the B-KPro does not eliminate the need for corneas from human donors [36]. Furthermore, a fresh corneal graft makes the B-KPro surgical procedure much easier and safer. Implanting the B-KPro device directly into the cornea of the patient would make the cornea more vulnerable to aseptic necrosis [37].

The most important aspect of the materials used for the B-KPro regards the interface between the optic stem and the surrounding tissues, i.e., biointegration. Making biointegration more stable and long-lasting can be the key to the success of these prosthetic devices. Most keratoprostheses, such as the B-KPro, are made entirely of rigid and transparent polymethyl methacrylate (PMMA) [40]. Due to the creation of the hole for the stem insertion, a narrow gap is observed between the polymeric stem and the adjacent corneal tissue, especially in inflamed situations. Clinical applications are currently underway to uncover the potential benefits of a titanium stem cap [40,41,42]. This coverage showed significantly improved corneal adhesion, thus leading to reduced exposure to pathogens or debris. In addition to the titanium coating, several attempts have been performed to coat the stem with inert biological substances to avoid infection. A hydroxyapatite coating would even be able to improve the healing of the surrounding cornea: Although very fragile to handle, it showed great promise. Other attempts included chemical functionalization with peptides capable of providing higher biointegration [40].

When preliminarily grafting the devices of the first generation, corneal tissue necrosis around the stem occurred; this phenomenon was due to the lack of nutrients and poor hydration from the anterior chamber of the eye. The large, solid back plate blocked necessary access to nutrients, resulting in aseptic cell death [40]. One of the first improvements to the backplate design was the addition of eight holes approximately 1.3 mm in diameter. These holes allowed for better contact of the cornea with the anterior chamber, thus permitting a higher hydration of the tissues and reducing the loss of structural integrity of the stroma with consequent fusion of the tissues [43,44]. The back plate was made of a variety of porous polymers, such as perforated grids of PMMA, nylon, ceramic, and Teflon. All these materials have been used in an attempt to overcome inflammatory complications and to improve the compatibility between the implant and surrounding tissues, making the implant able to integrate with the cornea [45]. Over the years, less rigid materials than PMMA have been introduced, but the success of these efforts has not been proven yet. The use of PMMA in the back plate can be replaced by medical-grade titanium: The lack of transparency could have a negative impact, as it shields stray light. A considerable advantage is due to the decrease in the incidence of retroprosthetic membrane formation from 30% to 13% when using a titanium plate combined with recent anti-inflammatory innovations [46,47], which occurs by far in 18–55% of cases [48]. However, the use of titanium or PMMA remains controversial, and the choice of the material mainly depends on surgeons’ experience [40].

To insert the backplate, the stem has a threaded design so that the surgeon can screw it manually. After several cases of loosening the back plate [40], another change in the device design was considered by inserting a titanium locking ring. This modification has several disadvantages, such as damage to the Descemet’s membrane and endothelium, resulting in prosthesis instability [43]. Currently, it is possible to apply treatments to the ring (i.e., gamma irradiation flooding) to allow for a more practical and safer internal assembly, even making the device to be stored longer in a small vial of fluid [36].

Another noteworthy prosthesis was introduced for the first time in 1963 by Benedetto Strampelli: It is the Osteo-Odonto-Keratoprosthesis (OOKP). It is one of the most long-lasting KPros [36], and its success is due to the biological components of the device [49]. Strampelli used a human tooth root and a part of the alveolar bone to make the cylindrical support. This device was further improved by G. Falcinelli in 1998 adding a larger biconvex optical lens as announced by Goossen et al. [50]. With this modification, the device is now known as the Modified Osteo-Odonto-Keratoprosthesis (MOOKP). It is worth mentioning that the implantation of a MOOKP device, and even an OOKP, requires a complex surgical procedure that can be performed only by very experienced surgeons [36]. 

It is worth mentioning that a few devices based on soft polymers have been translated from laboratory scale to human trials [51]. First, the Chirila KPro (also known as AlphaCor KPro) was implanted in humans in 1998 and received FDA approval in 2003 [52]; it is made of poly(2-hydroxyethyl methacrylate) (PHEMA) hydrogel, which is characterized by different water content in the central and peripheral parts of the device. The central part maintains optical transparency, and the peripheral skirt is prone to be repopulated by autologous keratocytes. The Legeais BioKpro III device represents an evolution of two previous models (I and II) and consists of two components: a 500 μm thick central silicone optic (5 mm diameter) and a surrounding disc (10 mm diameter) of opaque porous polytetrafluoroethylene [53]. This device was implanted in seven patients: Indeed, the keratoprosthesis failed in six patients due to partial or complete extrusion.

An exhaustive review of commercial and non-commercial keratoprotheses and their historical development has been published recently [54].

## 4. Replace or Regenerate?

Due to the recent advancements in tissue engineering approaches towards tissue/organ regeneration, major current shortcomings of corneal replacement could be bypassed by regenerating the cornea in vitro. In the following sections, biomaterials for cornea replacement and regeneration will be illustrated: They are intended to reduce the clinical dependence on human tissues from donors.

### 4.1. Biomaterials for Cornea Replacement and Restoration

A growing area of research aims at developing corneal substitutes, especially for low-risk cases, which are the majority of corneal transplants worldwide. Various materials have been used to replace the entire cornea or parts of it and to replicate its structure and function. Polymers, both natural and synthetic, are optimal candidates for producing corneal stroma matrices and substitutes. Natural polymers have the advantage of being “naturally” biocompatible; on the other hand, synthetic polymers allow for easier chemical manipulation and more versatile control of the mechanical characteristics to meet specific clinical needs.

Generally speaking, an ideal biomaterial should be biocompatible, resistant, transparent, non-immunogenic, refractive, permeable to nutrients and oxygen, resistant to neoangiogenesis [36]. For example, biopolymers from the extracellular matrix (ECM) have been investigated to mimic the corneal microenvironment. Theoretically, ECM components should be ideal for supporting cell growth (and also promoting tissue regeneration). In particular, collagen is the major component of the corneal stroma: Collagen types I, III, V form a complex network that provides considerable mechanical strength, which is difficult to replicate in the laboratory using collagen extracted from natural sources. Collagen is currently exploited for a wide range of biomedical applications in ophthalmology too [55]. In particular, collagen-based hydrogels are known to improve cornea regeneration because of their capacity to favor cell migration and colonization. Xeroudaki and coworkers developed a collagen-based porous hydrogel able to replace a substantial portion of damaged/diseased corneal stroma, obtaining the regeneration of host epithelium and nerves [56]. 

Different treatments have been applied to collagen-based hydrogels to increase their tensile strength. Collagen hydrogels are compressed plastically to increase density and cross-linked chemically with glutaraldehyde or genipin, physically using UV or dehydrothermal treatment, or enzymatically with transglutaminase. This can be a promising solution for patients at a high risk of graft failure, but it is currently suitable only for low-risk patients: The same kind of material (a bioengineered corneal implant produced with recombined human collagen type III, called RHCIII) implanted in rabbits with serious pathologies, presented neovascularization [57]. To reduce neovascularization for high-risk patients, RHCIII implants were modified to include the synthetic phospholipid methacryloyloxyethyl phosphorylcholine (MCP). The device was implanted in three patients with ulceration and reduced corneal integrity [57]. The implants improved the vision of two of them, and they all remained free from neovascularization after one year. A functional restoration of corneal integrity was also observed, with regeneration of both corneal epithelia and nerves, providing relief to all patients. In 2018, Islam et al. [58] implanted acellular corneal grafts made of recombined human collagen and MPC in an Anterior Lamellar Keratoplasty. Patients were unilaterally blind and at a high risk of graft failure. Three out of six patients gained significant improvements in vision and corneal stability [36].

A bio-orthogonally crosslinked hyaluronate–collagen hydrogel was proposed by Chen et al. to fill cornea defects in situ [59]: After a thorough characterization of the material, the authors applied it in a rabbit Anterior Lamellar Keratoplasty model. The results obtained were promising: The hydrogel, which is made of 97% water and is highly transparent, demonstrated excellent cytocompatibility and the ability to support epithelialization both in vitro and in vivo. More recently, an in situ-forming semi-interpenetrating polymer network (SIPN) hydrogel was investigated by the same group of authors in the same animal model [60]: The hydrogel demonstrated mechanical and optical properties similar to those of the native cornea, reducing stromal defect size compared with controls, and promoting multilayered epithelization. 

A few years before, the research group headed by Neil Lagali produced composite “core-and-skirt” collagen hydrogels with different degradation properties for corneal applications [61]. These hydrogels were characterized by a central part (transparent) as a stromal substitute and a peripheral skirt for delivering drugs and cells. They both possess adjustable transparency and degradability. These devices were tested in rabbits that underwent femtosecond laser-assisted intrastromal keratoplasties. The authors stated that the composites were biocompatible and able to integrate with the host cells, nerves, and collagen of the native stroma. 

Another injectable collagen-based biomaterial has been recently proposed to heal cornea perforations [62]. It is still transparent and injectable for up to 3 days after preparation and can be molded in situ; its mechanical properties can be controlled during the preparation, and its biocompatibility has been demonstrated in vitro with human corneal epithelial cells. 

Generally, hydrogels are very promising biomaterials for several clinical applications to repair and replace tissues and organs: They possess favorable characteristics such as tunable mechanical behavior, cytocompatibility, and, with specific regard to the cornea, optical properties [63].

### 4.2. Cells and Biomaterials for Cornea Regeneration

Biomaterial technology can offer new opportunities to overcome the limitations associated with current therapeutic approaches thanks to recent innovations and design evolution. Moreover, bioengineered corneal substitutes bypass the shortage of corneas from human donors. In the following paragraphs, scaffold-free, cell-free, and cell-based approaches will be briefly illustrated.

#### 4.2.1. Scaffold-Free Approach

Currently, stem cells are widely used for the regeneration of epithelium [64]; among others, there are pluripotent stem cells, epithelial stem cells, mesenchymal stem cells, and multipotent neural crest stem cells [65]. Many studies have also exploited stem cells from oral mucosa, bone marrow, adipose tissue, and amniotic membranes (Table 1). These types of stem cells are used for the regeneration of the corneal surface thanks to their ability to differentiate into cells with characteristics similar to corneal epithelial cells and also to secrete growth factors to promote corneal regeneration [4].

Cell sheets are used to regenerate a wide range of tissues, e.g., the esophagus, small blood vessels, heart tissue, and even corneas [66,67]. Although it can be an effective approach, tissue fragility is the main limitation: It makes transplantation difficult [4] and the removal of cell sheets complicated [36]. To overcome this drawback, various supports, such as temperature-sensitive polymeric substrates [68,69] or amniotic membranes [70,71], can be used.

Many in vitro and in vivo studies [72,73,74] have shown the potential of stem cells for ocular surface regeneration, confirming their differentiation capacities into cells with the characteristics of corneal epithelial cells.

#### 4.2.2. Cell-Free Scaffold

The use of corneal substitutes with innovative cell-free biomaterials would circumvent allogeneic cell-induced immune rejection, thus reducing the risk of graft failure and eliminating the need for chronic immunosuppression. Furthermore, the size of corneal implants can be adapted during manufacturing by modifying the shapes of the mold, for example, by modeling with a femtosecond laser, allowing for the creation of grafts with superior post-operative refractive results [75].

Decellularization is the process that allows for removing cells from tissues that can be used as scaffolds for new cells. Ideal corneal decellularization aims at removing all cellular and nuclear components, maintaining the transparency of the cornea and the structure of the ECM. Since cellular components of porcine cornea represent the major source of xenoantigens, decellularized corneas have the advantage of reducing the immune response after transplantation [76]. At present, decellularized corneas are one of the most promising solutions to replicate the complex structure and function of the cornea. Indeed, some hydrogels, which derive from ECM components, can be used for scaffold fabrication, but they lack the typical fibrillar organization and therefore exhibit different tensile strength properties. Decellularized corneas, however, retain the complex structure of corneal collagen. In particular, decellularized porcine corneas are commonly used, as they are easily procured and have anatomical characteristics similar to human ones. Anyway, it is necessary to eliminate epitopes that are extremely immunogenic for humans [36].

The decellularized porcine cornea is considered a medical device, although this issue is still debated. After decellularization, transparency is maintained, which, however, depends on the specific decellularization procedure applied. Decellularization methods are usually classified into three groups: chemical, physical, and biological. Chemical decellularization involves the application of detergents, including sodium dodecyl sulfate, Triton X-100 (In December 2012, the European Chemicals Agency (ECHA) included Triton X-100 in the Candidate List of substances of very high concern of the Registration, Evaluation, Authorization, and Restriction of Chemicals (REACH) Regulation, which addresses the production, import, and use of chemical substances and their potential impacts on human health and the environment. Indeed, a Triton X-100 degradation product has turned out to be ecotoxic, as it possesses hormone-like (estrogeno-mimetic) activity that may act on wildlife.), peracetic acid, formic acid, ammonium hydroxide, sodium chlorite, and ethylenediaminetetraacetic acid. Physical decellularization includes agitation, freeze–thaw, electrophoresis, high hydrostatic pressure, osmotic pressure, supercritical CO_2_, ultrasound, glycerol, and freeze-drying. In biological methods, mostly enzymes, including trypsin, dispase, phospholipase A2, human serum, and nuclease, are used. These methods can be used alone or in combination with each other [76]. 

From a general point of view, it is worth considering that increasing decellularization effectiveness often results in increased damage to the ECM. To check decellularization effectiveness, three criteria have to be satisfied: Intact cell nuclei have to be absent; the amount of double-stranded DNA has to be lower than 50 ng/mg of dry tissue; DNA residues have to be shorter than 200 base pairs [36,77]. An interesting comparison of the effectiveness of the most widely exploited decellularization protocols has been published in 2019 [78]. 

In the work presented by Polisetti et al. [79], sodium deoxycholate was used for decellularizing human corneas, which were then repopulated with primary human limbal epithelial cells, stromal cells, and melanocytes in vitro. A lamellar transplantation approach ex vivo was also performed. The authors claimed an effective removal of cellular and nuclear components while preserving ECM proteins, glycosaminoglycans, tissue structure, and optical transmission properties. The same group of authors investigated the decellularized human limbus as a scaffold for the transplantation of limbal epithelial progenitor cells [79]. They applied a similar decellularization protocol (sodium deoxycholate and deoxyribonuclease I) and evaluated the efficiency of decellularization and its effects on ECM composition and structure. They confirmed that the decellularization protocol is effective in removing cellular and nuclear material without alteration of the native ECM. The acellular scaffold also demonstrated good biocompatibility.

Xenogeneic corneas were also studied in order to produce acellular matrix for tissue regeneration. For example, porcine corneas were decellularized with hydrostatic pressure [80], a hypotonic buffer and SDS [81], hypertonic saline and nitrogen [82], and with a combination of detergent (Triton X-100) and a freeze-drying process [83], and then characterized in vitro and in vivo.

#### 4.2.3. Cell-Based Scaffold

A potential candidate biomaterial for a cell-based scaffold should be made of stable and easily processable polymer, which may have initial cell adhesion properties; furthermore, it should allow for the detachment of a cohesive monolayer when required [82]. Tissue engineering for corneal regeneration involves the creation of constructs (scaffolds) suitable for culturing cells to generate a phenotype similar to that of the native cornea [36]. Many studies proposed engineered matrices with the aim of regenerating corneal epithelium and imitating its native microenvironment [75]. For this purpose, 3D structures made with different materials can be used [36]. The choice of appropriate biomaterial is of paramount importance in tissue engineering strategies; their biocompatibility is the most important feature. 

For epithelial regeneration, Momenzadeh et al. [84] used stem cells derived from the human eyelid fat, cultured on a layer of cross-linked nanofibrous gelatin. The results showed an increase in stem cell viability and high expression of cytokeratin 3 and 12. This gelatinous structure is still under investigation because it represents a suitable alternative to the amniotic membrane.

Another type of scaffold requires the use of cross-linked collagen hydrogel, with natural transparency and adequate mechanical properties, as an engineered matrix for corneal epithelium [4]. Implants based on recombined human collagen type III (RHCIII) showed stable integration without the need for immunosuppression, successfully mimicking the ECM of the cornea, promoting the ingrowth of corneal epithelial cells, nerves, and stromal keratocytes [75].

It has been demonstrated [74,85] that limbal stem cells cultured on scaffolds with aligned fibers of poly (ester urethane) urea (PEUU) showed differentiation capacity into keratocytes, with a high expression of specific markers and a deposition of a dense, uniformly aligned collagen matrix. The topographic signals of the scaffold are crucial for the alignment of limbal stem cells and their functional differentiation into corneal keratocytes, resulting in the formation of transparent corneal tissue. The study also showed how limbal cells cultured on scaffolds with non-aligned PEUU fibers produce a dense matrix but with non-aligned collagen fibers: This morphological disorder compromises the transparency of the engineered cornea. 

Silk fibroin is another promising biomaterial for the production of corneal scaffolds [6]: It is non-immunogenic and allows for easy production of high-resolution patterns via lithographic techniques [36]. In order to improve the structural integrity of the silk-based scaffolds, their cell adhesion/proliferation, and cell migration, several studies managed to blend fibroin-based scaffolds with several other biomaterials, including arginyl–glycyl–aspartic acid (RGD) peptide [86], poly-D-lysine (PDL) [87], aloe vera [88], β-carotene [89], lysophosphatidic acid [90], chitosan [91], and collagen obtaining significant results [48]. These characteristics make silk fibroin an ideal material to use as a substrate for the construction of the cornea and other tissues [36].

Regarding the corneal endothelial cells, the most successful synthetic polymer to date is the temperature-responsive polymer poly(N-isopropylacrylamide, PIPAAm), which has already been used clinically for corneal epithelial cell expansion [64]. As highlighted in the review published by Navaratnam et al., several studies have shown that PIPAAm can support important cell structures (i.e., the Na^+^/K^+^-ATPase pump) and the morphology of CECs with the presence of microvilli and cellular interconnections [65]. Moreover, it allows for the detachment of a single cellular cohesive monolayer when required.

## 5. Future Perspectives

Scientific research is running fast in the field of biomedical innovations, so it is quite difficult to draw the lines of the future evolution of cornea repair and replacement, and to hypothesize the timing for the clinical translation of current results. Anyway, it is worthy to mention two promising approaches that surely appear attractive: the bioprinting technique and the xenotransplantation, which will be briefly illustrated in the following paragraphs.

### 5.1. Bioprinting

**Figure 7:**
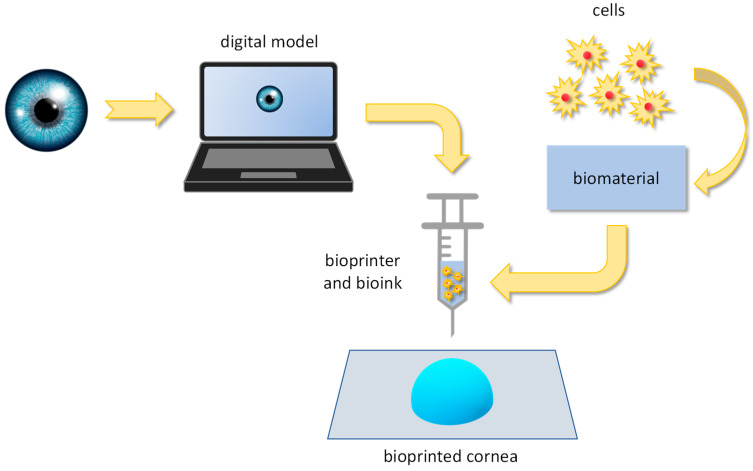
The process for bioprinting the human cornea: from the digital model to the printed 3D structure.

Bioprinting is the 3D printing technique used for the production of three-dimensional biological tissues through the layer-by-layer deposition of a bioink that contains living cells: It is well-acknowledged for its ability to hierarchically assemble biological structures [92,93]. Three-dimensional bioprinting shows promising features in generating bioengineered tissues, such as the human cornea. Basically, a digital model of the target tissue/organ is created from bioimages (i.e., CT and MRI scans): From the model, the printing path is defined to guide the bioprinter depositing the bioink (a biomaterial enriched with cells) layer by layer (Figure 7). Up to now, several bioprinting techniques are available; they mainly differ on the basis of the mechanism for bioink deposition: extrusion bioprinting, laser-assisted bioprinting, inkjet bioprinting, stereolithography, and digital light processing.

Research on bioprinted corneas has sparked great interest due to the wide range of materials and cells that can be used (Table 2). Thanks to the growing technological progress in this field, it will likely reach a level of clinical functionality that will allow for overcoming the limited availability of tissues from human donors, also eliminating the problem due to the possible transmission of infectious diseases [94]. 

A bioprinted cornea has to meet severe geometrical, biological, optical, and mechanical requirements [106]. Thus, the choice for an adequate bioink is critical: It is quite difficult, but fundamental, to find a biomaterial possessing excellent optical transparency, sufficient mechanical resistance, and, at the same time, good biological compatibility and integrability. Among other biomaterials, hydrogels have received extensive attention thanks to their unique features [107]. Hydrogels can be defined as highly hydrophilic three-dimensional polymeric networks able to entrap large amounts of water (or biological fluids) without dissolving. A very well-organized systematic review on hydrogels for bioprinting was published in 2020 [108]: It focuses on hydrogels obtained from both natural and synthetic components. Natural materials include, for example, alginate (even in the form of oxidized and methacrylated alginate), gelatin, agarose, cellulose, and collagen. As the main component of the corneal stroma, collagen type I is suitable for producing bioprinted corneal substitutes [109,110]. In the work presented by Duarte Campos et al. [110], corneal stromal keratocytes were embedded in collagen-based bioinks used to fabricate 3D biomimetic models, which were evaluated in vitro: Corneal stromal equivalents with optical properties similar to native tissue were achieved. A major challenge concerns the precise control of the collagen concentration to obtain the necessary mechanical strength. To face this issue, some authors experimented with different collagen concentrations [109] to optimize the mechanical properties of the engineered constructs and their transparency as well. Sometimes, collagen was used in combination with alginate [111]. Alginate is another natural polymer obtained from brown algae, and it has been widely studied for several biomedical applications due to its biocompatibility, low toxicity, and relatively low cost. Alginate can be also used to form composite materials [112].

Agarose is another promising hydrogel: it is a linear polymer that is generally extracted from some red algae. Agarose can be used alone or in combination with other biomaterials [113]. Other natural polymers, such as hyaluronic acid and chitosan, are used in several biomedical applications and also for the production of structures mimicking the corneal matrix [114,115,116]. 

Synthetic polymers are suitable for the formulation of bioinks due to their tunable characteristics, such as their rheological features, degradability, gelation mechanisms, and biological properties. In particular, synthetic polymers allow for structural modification and chemical functionalization of naturally derived hydrogels [117]. Synthetic biomaterials can include polyethylene glycol (PEG), pluronic (a poloxamer), polylactic acid (PLA), polycaprolactone (PCL), and polylactic-co-glycolic acid (PLGA) [118]. Better biomechanical resistance can be usually achieved by formulating combinations of two (or more) different biomaterials [119].

Whatever the material of choice, bioinks have to maintain the structure of the bioprinted construct, promote cell growth and spreading, and support their maturation into the appropriate tissues [119].

### 5.2. Xenoimplants

The term “xenoimplant” refers to the procedure (xenotransplantation) that involves the transplantation into humans of cells, tissues, or organs from a nonhuman animal source. Generally speaking, this procedure has great potential in overcoming the “organ shortage”, but it presents at least two critical drawbacks: the risk of the transmission of infectious diseases (from animal to humans) and the risk of immune rejection. Indeed, immune rejection and the need for immunosuppression therapy remain the main barriers to xenoimplants [74]. 

Which animal is the best source for xenotransplantation? Primates have higher similarities to humans in anatomy, physiology, and immunology, but pigs are preferred due to many advantages [120]: Pigs reach reproductive maturity and maximum size much faster than primates, and their litter size is significantly higher. Moreover, the costs for the animals’ maintenance are lower, and genetic engineering and cloning techniques are more experienced in pigs. In order to prevent the immunological response, huge progress has been made by genetically modifying the animal sources, i.e., knocking out pigs for the alpha-1,3-galactosyltransferase gene. Indeed, the alpha-Gal epitope, absent in humans, is known to be the key xenoantigen eliciting rejection. 

In general, it is necessary to know the mechanism underlying the immune rejection of a xenogeneic tissue/organ in order to choose the most appropriate genetically modified pigs for corneal xenografts [74]. Although the mechanisms behind xenografts hyperacute rejection are well-understood, those of acute rejection are not. Furthermore, unlike allografts, there is a lack of data on standardized predictive and diagnostic markers, which allow for greater monitoring of xenografts [121]. Recently, a gene editing technology using CRISPR/Cas9 was announced: By manipulating multiple genes in a short period of time, it is possible to create genetically modified pigs that are less immunogenic and exhibit a lower risk of xenozoonosis [122]. This technology employs DNA endonucleases, CRISPR and Cas9, to perform site-specific mutagenesis [123]. Therefore, it is possible to overcome the rejection associated with the presence of the α-Gal epitope that is removed [121]. This new important solution is a great step forward in clinical trials and also arouses the interest of financiers for reinvigorating research on xenotransplantation [74]. 

In 2022, the International Human Xenotransplantation Inventory reported the application of acellular porcine cornea in 25 patients to treat fungal keratitis: In 20 patients, the grafts remained transparent at 1 year of follow-up [124]. For an exhaustive review on cornea xenotransplantation, we suggest the paper published by Yoon, Choi, and Kin in 2021 [74]: Besides the state-of-the-art on xenotransplantation, it describes xenogeneic rejection mechanisms and current immunosuppression therapies, preclinical results and safety data, and the regulatory framework for clinical trials. 

## 6. Conclusions

Bibliographic research offers a huge number of scientific papers on the cornea. Using the keyword “cornea”, PubMed returns more than 95,000 references: The oldest one dates to 1799, and it regards “the use of an instrument for cutting the cornea, in the operation of extracting a cataract” [125]. It was written by Sir Anthony Carlisle, who was appointed surgeon at the Westminster Hospital (London, UK) in 1793, where he remained for 47 years. A few other papers were issued through half of the 1900s: The vast majority of the references start from 1990, and almost 70,000 papers were published between 2000 and 2024: One of the most recent papers describes the cornea as “one of the pioneering areas of regenerative medicine” [126], opening hope for new and more effective therapeutic treatments with the aim of repairing/replacing damaged/diseased corneas with engineered constructs that are mainly based on stem cells.

## Figures and Tables

**Table 1:** Stem cells from corneal and non-corneal sources.

Corneal Stem Cells	Non Corneal Stem Cells	
Limbal epithelial	Pluripotent	Embryonic
Limbal mesenchymal		iPS
	Epithelial	Oral mucosa
		Hair follicle
		Epidermis
		Amniotic membrane
	Mesenchymal	Bone marrow
		Adipose derived
		Amniotic membrane
		Placenta
		Umbilical cord
	Neural crest	Dental pulp

**Table 2:** Characteristics of the most widely used 3D printing techniques.

	Droplet-Based (DBB)			Extrusion-Based (EBB)	Vat Photopolymerization (VP)			
Typology	Thermal	Piezoelectric	Micro-Valves	EBB	SLA	DLP	CLIP	Volumetric Printing
Print rate	Fast (1–10^4^ droplets/s) [95,96,97] 10^5^ droplets/s [98] 30 kHz [99]	Fast (1–10^4^ droplets/s) [95,96,97] 10^5^ droplets/s [98] 30 kHz [99]	kHz [99]	Slow [95,100] 10–700 mm/s [98]	Fast [95,96,101]	Fast [96], Very fast [99]	Very fast (25–100 mm/h) [99]	Extremely fast (seconds) [102]
% Cell survival	>85% [95,96,97,98]	NT [99]	>85% [95,96,97,98]	80–90% [96,98] 40–80% [95,97]	>90% [96], >85% [95]	>SLA [96]	NT [99]	
Cellular density (cells/mL)	<10^6^ [95,96,99]	NT [99]	<10^6^ [95,96,99]	>10^8^ [95,96,98]	<10^8^ [95,96]	<10^8^ [96]	NT [99]	
Range of viscosity	3.5–12 mPa/s [95,96,97] <10 mPa/s [98] <15 mPa/s [99]	3.5–12 mPa/s [95,96,97] <10 mPa/s [98] <15 mPa/s [99]	3.5–12 mPa/s [95,97] <10 mPa/s [98] <200 mPa/s [99]	30–6·10^7^ mPa/s [95,96,97,98]	- [95,96]	10–5000 mPa/s [99]	10–5000 mPa/s [99]	<90,000 mPa/s [99]
Resolution	75 µm [96] High [95,100] 10–50 µm [98] 50–500 µm [99]	75 µm [96] High [95,100] 10–50 µm [98] 50–500 µm [99]	75 µm [96] High [95,100] 10–50 µm [98] 50–500 µm [99]	200–1000 µm [98] Good [95,97] Low [100] 100–600 µm [99]	60–150 µm [96] Very high [100,101,103,104] >5 µm [102]	25–50 µm [96] >5 µm [102] 25–150 µm [99]	25–150 µm [99]	>100 µm [102] 25–150 µm [99]
Vertical resolution	Low [95]	Low [95]	Low [95]	High [95]	50–150 µm [101]	25–100 µm [99]	≈10 µm [99]	
Mechanical properties	Low [99,104] Moderate [103]	Low [99,104] Moderate [103]	Low [99,104] Moderate [103]	Low [100]	Low [100,101] Moderate [103]	Very good [99]	Very good [99]	Very good [99]
Scaffold dimension	cm [99]	cm [99]	cm [99]	cm [99]	Wide range ^k^	cm [99]	cm [99]	cm [99]
Cost	Low [95,97,98,104] 5000 $ [99]	>Thermal [99] Low [95,98]	5000 $ [99]	30 k–250 k $ [99]	Medium [101,103] 3.5 k–5 k $ [105]	30 k–50 k $ [99]	30 k–50 k $ [99]	30 k–50 k $ [99]
Risks and drawbacks	Nozzle obstruction; cellular damage at 15–25 kHz [97]; limited pore size and dissolution if organic solvents are used [100]; thermal technology possibly oncogenic [99]			Mechanical or thermal stress at cellular level [98,100]	Require long post-printing treatments [104]	Risk of cellular damage due to UV rays or photo-initiators [99]		Risk of cellular damage due to UV rays or photo-initiators [99]; High density require a lot of software changes [102]
